# Crystal structure of bis­[μ-*N*-(η^2^-prop-2-en-1-yl)piperidine-1-carbo­thio­amide-κ^2^ *S*:*S*]bis­[(thio­cyanato-κ*N*)copper(I)]

**DOI:** 10.1107/S2056989020013146

**Published:** 2020-10-06

**Authors:** Takeshi Tanaka, Yukiyasu Kashiwagi, Masami Nakagawa

**Affiliations:** a Osaka Research Institute of Industrial Science and Technology, 2-7-1 Ayumino, Izumi, Osaka 594-1157, Japan; b Osaka Research Institute of Industrial Science and Technology, 1-6-50 Morinomiya, Joto-ku, Osaka 536-8553, Japan

**Keywords:** crystal structure, Cu^I^ dimer, thio­urea, N—H⋯S inter­action, C—H⋯S inter­action, *η*^2^-*π*-allyl coordination

## Abstract

The title crystalline compound, [Cu_2_(NCS)_2_(C_9_H_16_N_2_)_2_], was obtained from the reaction of copper(I) thio­cyanate (CuSCN) with (*N*-prop-2-en-1-yl)piperidine-1-carbo­thio­amide as a chelating and bridging thio­urea ligand in chloro­benzene. The Cu_2_S_2_ core of the dimeric mol­ecule is situated on a crystallographic inversion centre. The copper atom is coordinated by a thio­cyanate nitro­gen atom, each sulfur atom of the two thio­urea ligands, and the C=C double bond of the ligand in a distorted tetra­hedral geometry. The dimers are linked by N—H⋯S hydrogen bonds, forming a network extending in two dimensions parallel to (100).

## Abstract 2

The crystal structure of the title compound consists of a dimeric Cu^I^ complex possessing a Cu_2_S_2_ core and contains thio­cyanate anions and allyl­thio­urea derivatives as chelating and bridging ligands. The dimeric Cu^I^ complexes are linked by N—H⋯S hydrogen bonds, forming a network extending in two dimensions parallel to (100).

## Chemical context   

Thio­urea and its derivatives, *N*-substituted thio­urea and *N*, *N′*-disubstituted thio­urea, are well-known ligands to copper ions, such as for their structural relatedness of proteins in bioinorganic chemistry and controlling redox potentials of copper ions in electrochemistry. Recently, copper–thio­urea complexes [Cu(tu)s] have been investigated as electronic materials, for precursors of copper sulfide to be applied as semiconductors (Shamraiz *et al.*, 2017 ▸; Sarma *et al.*, 2019 ▸; Patel *et al.*, 2019 ▸), photocatalysts (Tran *et al.*, 2012 ▸; Pal *et al.*, 2015 ▸), and sensors (Liu & Xue, 2011 ▸; Sabah *et al.*, 2016 ▸; Sagade & Sharma, 2008 ▸). Cu(tu)s have also been used as a component of the precursor ink for forming CuIn(S, Se) as photo-absorbing layers in solar cells (Uhl *et al.*, 2016 ▸). The solubility of Cu(tu)s in non-polar solvents is a potentially important property for their application as electronic materials. In order to synthesize a hydro­phobic Cu(tu)s, we developed an allyl and a piperidinyl group bearing thio­urea, (*N*-prop-2-en-1-yl)piperidine-1-carbo­thio­amide, as a hydro­phobic bidentate ligand and report here the crystal structure of the title non-ionic Cu^I^ complex containing thio­cyanates as coordinating anions.
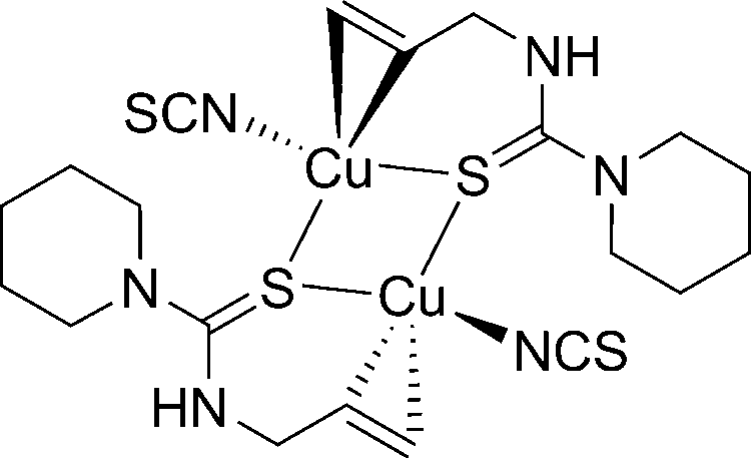


## Structural commentary   

**Figure 1:**
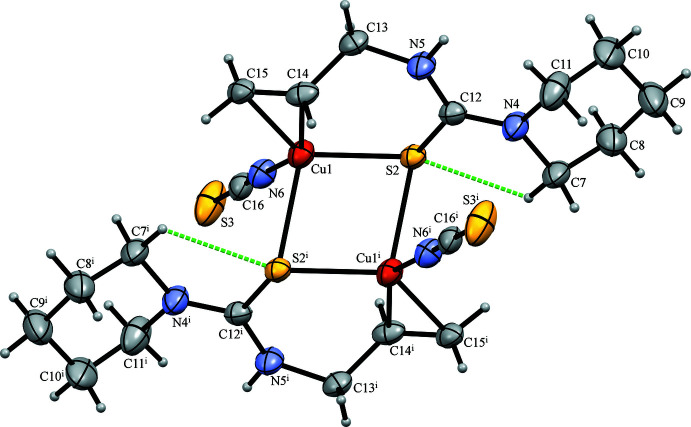
The mol­ecular structure of the title compound, with atom labelling. Displacement ellipsoids are drawn at the 50% probability level. H atoms are represented by spheres of arbitrary radius. The hydrogen bonds are shown as green dashed lines. [Symmetry code: (i) −*x* + 1, −*y* + 1, −*z* + 1].

The mol­ecular structure of the title compound possessing a Cu_2_S_2_ central core is shown in Fig. 1 ▸. The dimeric mol­ecule is situated on a crystallographic inversion centre. Selected geometric parameters are shown in Table 1 ▸. The coordination about the Cu atom can be described as distorted tetra­hedral containing N6, S2, S2^i^, and *Cg*1 [*Cg*1 is the mid-point of C14 and C15; symmetry code: (i) −*x* + 1, −*y* + 1, −*z* + 1]. The four-coordinate geometry index, *τ*_4_ = [360° - (*α* + *β*)] / 141°, evaluated from the two largest angles (*α* < *β*), which has ideal values of 1 for a tetra­hedral and 0 for a square-planar geometry (Yang *et al.*, 2007 ▸), is equal to 0.83. The Cu⋯Cu^i^ separation in the dimer is 3.1180 (6) Å. The C14=C15 double bond is *η* ^2^-*π*-coordinated to Cu, the bond being elongated to 1.351 (5) Å. The N atom of the piperidine ring (N4) shows no pyramidalization, with a displacement of 0.041 (3) Å from the plane of the bonded C atoms (C7, C11 and C12). The piperidine ring adopts a chair conformation with puckering parameters: *Q* = 0.573 (4), *θ* = 176.3 (4), and *φ* = 153 (6) (Cremer & Pople, 1975 ▸). There is one intra­molecular inter­action, C7—H7*B*⋯S2, generating an *S*(5) ring motif (Fig. 1 ▸ and Table 2 ▸). In comparison, the crystal structure of bis­(aceto­nitrile)­bis­(*η* ^2^-*N*-allyl­thio­urea)dicopper(I) dinitrate [Cu_2_(atu)_2_(CH_3_CN)_2_](NO_3_)_2_, a cationic analogue of the title compound with aceto­nitrile instead of thio­cyanate and without the piperidine ring, shows a similar geometry around copper but has no crystallographic inversion centre because of the asymmetric packing of the nitrate anions [Cambridge Structural Database (CSD) refcode RENNON; Filinchuk *et al.*, 1996 ▸].

## Supra­molecular features   

**Figure 2:**
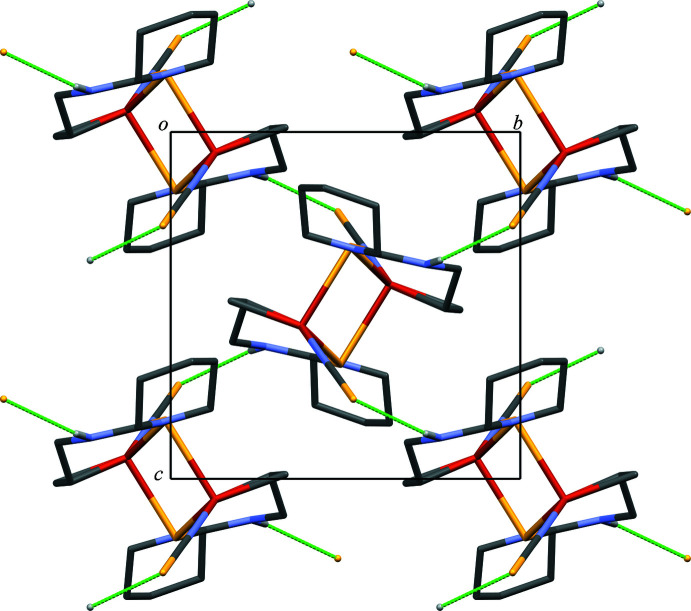
A packing diagram of the title compound viewed along the *a* axis, *i.e.* a top view of the two-dimensional network. The N—H⋯S hydrogen bonds are shown as green dashed lines. H atoms not involved in the inter­actions were omitted for clarity.

**Figure 3:**
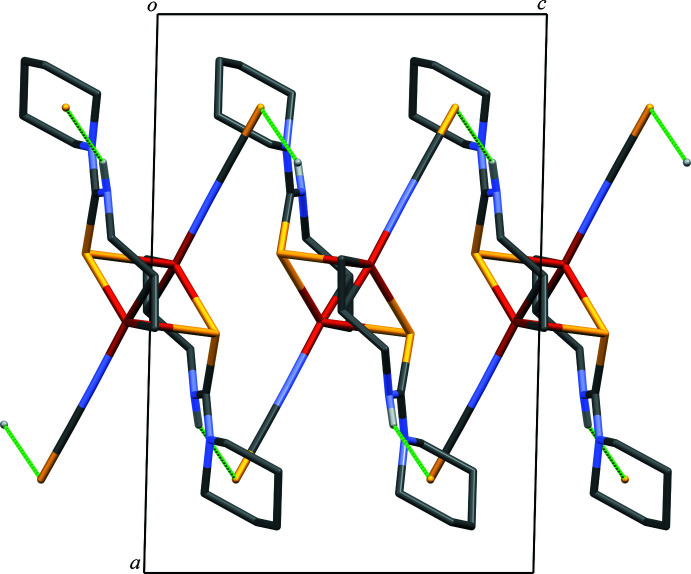
A packing diagram of the title compound viewed along the *b* axis, *i.e.* a side view of the two-dimensional network. The N—H⋯S hydrogen bonds are shown as green dashed lines. H atoms not involved in the inter­actions were omitted for clarity.

In the crystal, the dimers are linked by N—H⋯S hydrogen bonds [N5—H5⋯S3^ii^; symmetry code: (ii) −*x* + 1, *y* + 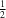, −*z* + 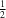], forming a network extending in two dimensions parallel to (100) (Fig. 2 ▸, Fig. 3 ▸, and Table 2 ▸). There is no significant inter­action between two-dimensional networks. In contrast, the crystal structure of [Cu_2_(atu)_2_(CH_3_CN)_2_](NO_3_)_2_ exhibits a complementary C—H⋯S inter­action between discrete copper dimers forming a dimer of dimeric structures (RENNON; Filinchuk *et al.*, 1996 ▸). The discrete copper dimer exhibits six N—H⋯O inter­actions to the surrounding six nitrate anions.

## Database survey   

A search of the CSD (Version 5.41, update of August 2020; Groom *et al.*, 2016 ▸) using *ConQuest* (Bruno *et al.*, 2002 ▸) for compounds containing the 1-allyl­thio­urea skeleton gave 892 hits, and for those containing the thio­urea derivatives as ligands gave 945 hits of Cu complexes. The crystal structures of the ligand of the title compound, (*N*-prop-2-en-1-yl)piperidine-1-carbo­thio­amide, itself and its metal complexes have not been reported. A survey for a Cu complex containing the 1-allylthio­urea fragment as a *κS*-coordination ligand reveals 53 examples, which includes six examples of *η* ^2^-*π*-coordination of an allyl group to Cu. All of these six examples are Cu^I^ complexes, which comprise four coordination polymers of 4-allyl-semicarbazide as ligands (Mel’nyk *et al.*, 2001 ▸, 2011 ▸; Olijnik *et al.*, 2011 ▸), one coordination polymer of 1,3-di­allyl­thio­urea as ligand (BOGNUH; Vakulka *et al.*, 2007 ▸), and one discrete centrosymmetric dimer of 1-allyl­thio­urea as ligand (RENNON; Filinchuk *et al.*, 1996 ▸).

## Synthesis and crystallization   

To a chloro­benzene solution (2.5 mL) containing copper(I) thio­cyanate (CuSCN, 122 mg, 1.0 mmol) and allyl iso­thio­cyanate (298 mg, 3.0 mmol) in a 20 mL capped screw-tube bottle was slowly added piperidine (171 mg, 2.0 mmol) at 373 K under air and the mixture was stirred for 5 minutes. After that, it was left at room temperature. The pale-white precipitate formed in the bottle, and gradually changed to a pale-white solid containing single crystals. The mixture was filtered after 5 days to give a pale-white solid containing single crystals (267 mg, 0.87 mmol, 87%). Single crystals suitable for X-ray crystallographic analysis were selected in the product. Analysis calculated for (C_10_H_16_CuN_3_S_2_)_2_: C, 39.26; H, 5.27; N, 13.74; S, 20.96. Found: C, 38.72; H, 4.78; N, 13.59; S, 20.28.

## Refinement   

Crystal data, data collection and structure refinement details are summarized in Table 3 ▸. Atoms H14, H15*A*, and H15*B* were located in a difference-Fourier map and refined freely, considering the influence of the coordination of the ethenyl group to Cu^I^. H11*A* and H11*B* were also located in the difference-Fourier map and refined freely, because the distance between intramolecular H11*B* and H5 in the neighbouring mol­ecule was abnormally short in the riding model. Other C-bound H atoms were placed in geometrically calculated positions (C—H = 0.99 Å) and refined as part of a riding model with *U*_iso_(H) = 1.2*U*_eq_(C). The N-bound H5 atom was located in the difference-Fourier map but was refined with a distance restraint of N—H = 0.86±0.01 Å, and with *U*_iso_(H) set to 1.2*U*_eq_(N).

## Supplementary Material

Crystal structure: contains datablock(s) I. DOI: 10.1107/S2056989020013146/yz2001sup1.cif

Structure factors: contains datablock(s) I. DOI: 10.1107/S2056989020013146/yz2001Isup2.hkl

CCDC reference: 2034488

Additional supporting information:  crystallographic information; 3D view; checkCIF report

## Figures and Tables

**Table 1:** Selected geometric parameters (Å, °)

Cu1—S2	2.2835 (8)	Cu1—C15	2.095 (3)
Cu1—S2^i^	2.6491 (8)	Cu1—*Cg*1	1.969
Cu1—N6	1.924 (3)	N5—H5	0.856 (10)
Cu1—C14	2.068 (3)	C14—C15	1.351 (5)
			
S2—Cu1—S2^i^	101.98 (3)	C14—Cu1—C15	37.86 (13)
N6—Cu1—S2	107.15 (8)	C15—Cu1—S2	130.85 (10)
N6—Cu1—S2^i^	97.44 (8)	C15—Cu1—S2^i^	101.56 (10)
N6—Cu1—C14	147.71 (13)	Cu1—S2—Cu1^i^	78.02 (3)
N6—Cu1—C15	111.75 (13)	*Cg*1—Cu1—S2	113.57
C14—Cu1—S2	95.68 (9)	*Cg*1—Cu1—S2^i^	101.31
C14—Cu1—S2^i^	99.83 (9)	*Cg*1—Cu1—N6	129.88

Symmetry code: (i) 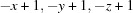.

**Table 2:** Hydrogen-bond geometry (Å, °)

*D*—H⋯*A*	*D*—H	H⋯*A*	*D*⋯*A*	*D*—H⋯*A*
N5—H5⋯S3^ii^	0.86 (2)	2.60 (3)	3.375 (3)	151 (3)
C7—H7*B*⋯S2	0.99	2.48	3.028 (3)	114

Symmetry code: (ii) 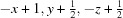.

**Table 3:** Experimental details

Crystal data	
Chemical formula	[Cu_2_(NCS)_2_(C_9_H_16_N_2_S)_2_]
*M*_r_	611.83
Crystal system, space group	Monoclinic, *P*2_1_/*c*
Temperature (K)	173
*a*, *b*, *c* (Å)	13.9881 (5), 9.8220 (4), 9.7446 (4)
β (°)	91.391 (6)
*V* (Å^3^)	1338.43 (9)
*Z*	2
Radiation type	Mo *K*α
μ (mm^−1^)	1.92
Crystal size (mm)	0.15 × 0.15 × 0.1

Data collection	
Diffractometer	Rigaku R-AXIS RAPID
Absorption correction	Multi-scan (*ABSCOR*; Higashi, 1995 ▸)
*T*_min_, *T*_max_	0.747, 1.000
No. of measured, independent and observed [*I* > 2σ(*I*)] reflections	12737, 3071, 2516
*R*_int_	0.041
(sin θ/λ)_max_ (Å^−1^)	0.649

Refinement	
*R*[*F* ^2^ > 2σ(*F* ^2^)], *wR*(*F* ^2^), *S*	0.045, 0.098, 1.08
No. of reflections	3071
No. of parameters	168
No. of restraints	1
H-atom treatment	H atoms treated by a mixture of independent and constrained refinement
Δρ_max_, Δρ_min_ (e Å^−3^)	0.61, −0.40

Computer programs: *RAPID-AUTO* (Rigaku, 2006 ▸), *SHELXT* 2018/2 (Sheldrick, 2015*a* ▸), *SHELXL2018/3* (Sheldrick, 2015*b* ▸), *PLATON* (Spek, 2020 ▸), *Mercury* (Macrae *et al.*, 2020 ▸), *OLEX2* (Dolomanov *et al.*, 2009 ▸) and *publCIF* (Westrip, 2010 ▸).

## supplementary crystallographic information

### Crystal data

**Table:**

[Cu_2_(NCS)_2_(C_9_H_16_N_2_S)_2_]	*F*(000) = 632
*M**_r_* = 611.83	*D*_x_ = 1.518 Mg m^−^^3^
Monoclinic, *P*2_1_/*c*	Mo *K*α radiation, λ = 0.71075 Å
*a* = 13.9881 (5) Å	Cell parameters from 9884 reflections
*b* = 9.8220 (4) Å	θ = 5.0–55.0°
*c* = 9.7446 (4) Å	µ = 1.92 mm^−^^1^
β = 91.391 (6)°	*T* = 173 K
*V* = 1338.43 (9) Å^3^	Block, clear colourless
*Z* = 2	0.15 × 0.15 × 0.1 mm

### Data collection

**Table:**

Rigaku R-AXIS RAPID diffractometer	3071 independent reflections
Radiation source: sealed X-ray tube	2516 reflections with *I* > 2σ(*I*)
Graphite monochromator	*R*_int_ = 0.041
ω scans	θ_max_ = 27.5°, θ_min_ = 2.5°
Absorption correction: multi-scan (ABSCOR; Higashi, 1995)	*h* = −18→17
*T*_min_ = 0.747, *T*_max_ = 1.000	*k* = −12→12
12737 measured reflections	*l* = −12→12

### Refinement

**Table:**

Refinement on *F*^2^	Primary atom site location: dual
Least-squares matrix: full	Hydrogen site location: mixed
*R*[*F*^2^ > 2σ(*F*^2^)] = 0.045	H atoms treated by a mixture of independent and constrained refinement
*wR*(*F*^2^) = 0.098	*w* = 1/[σ^2^(*F*_o_^2^) + (0.0406*P*)^2^ + 1.6287*P*] where *P* = (*F*_o_^2^ + 2*F*_c_^2^)/3
*S* = 1.08	(Δ/σ)_max_ = 0.001
3071 reflections	Δρ_max_ = 0.61 e Å^−^^3^
168 parameters	Δρ_min_ = −0.40 e Å^−^^3^
1 restraint	

### Special details

**Table:**

Geometry. All esds (except the esd in the dihedral angle between two l.s. planes) are estimated using the full covariance matrix. The cell esds are taken into account individually in the estimation of esds in distances, angles and torsion angles; correlations between esds in cell parameters are only used when they are defined by crystal symmetry. An approximate (isotropic) treatment of cell esds is used for estimating esds involving l.s. planes.

### Fractional atomic coordinates and isotropic or equivalent isotropic displacement parameters (Å^2^)

**Table:**

	*x*	*y*	*z*	*U*_iso_*/*U*_eq_	
Cu1	0.55125 (3)	0.62670 (4)	0.43953 (4)	0.03267 (13)	
S2	0.42819 (5)	0.51488 (7)	0.33025 (7)	0.02780 (17)	
S3	0.83263 (6)	0.47774 (11)	0.22981 (13)	0.0594 (3)	
N4	0.23866 (19)	0.5307 (3)	0.3387 (3)	0.0402 (6)	
N6	0.66753 (18)	0.5688 (3)	0.3556 (3)	0.0353 (6)	
N5	0.31829 (18)	0.7313 (3)	0.3819 (3)	0.0376 (6)	
H5	0.2655 (15)	0.774 (3)	0.369 (4)	0.045*	
C14	0.4691 (2)	0.7864 (3)	0.5090 (3)	0.0346 (7)	
C16	0.7358 (2)	0.5304 (3)	0.3044 (3)	0.0331 (7)	
C12	0.3206 (2)	0.5985 (3)	0.3524 (3)	0.0301 (6)	
C15	0.5614 (2)	0.8269 (3)	0.5125 (4)	0.0342 (7)	
C13	0.4006 (2)	0.8228 (3)	0.3952 (3)	0.0367 (7)	
H13A	0.377117	0.916542	0.410448	0.044*	
H13B	0.435145	0.822461	0.307731	0.044*	
C7	0.2322 (2)	0.3865 (3)	0.2969 (4)	0.0448 (9)	
H7A	0.197211	0.334276	0.366632	0.054*	
H7B	0.297289	0.347654	0.291007	0.054*	
C8	0.1816 (3)	0.3743 (4)	0.1611 (4)	0.0510 (9)	
H8A	0.174400	0.276834	0.137225	0.061*	
H8B	0.220513	0.417941	0.089947	0.061*	
C11	0.1427 (3)	0.5915 (4)	0.3497 (5)	0.0548 (11)	
C10	0.0912 (3)	0.5872 (4)	0.2135 (5)	0.0608 (12)	
H10A	0.126101	0.642923	0.146529	0.073*	
H10B	0.026342	0.626044	0.222292	0.073*	
C9	0.0836 (3)	0.4406 (4)	0.1618 (5)	0.0625 (12)	
H9A	0.041038	0.388028	0.221843	0.075*	
H9B	0.055244	0.439964	0.067737	0.075*	
H15A	0.600 (2)	0.820 (3)	0.596 (3)	0.026 (8)*	
H15B	0.591 (3)	0.872 (4)	0.437 (4)	0.048 (11)*	
H14	0.439 (2)	0.754 (4)	0.592 (4)	0.048 (10)*	
H11A	0.096 (3)	0.536 (4)	0.409 (4)	0.046 (10)*	
H11B	0.148 (3)	0.677 (5)	0.384 (4)	0.065 (13)*	

### Atomic displacement parameters (Å^2^)

**Table:**

	*U*^11^	*U*^22^	*U*^33^	*U*^12^	*U*^13^	*U*^23^
Cu1	0.0320 (2)	0.0261 (2)	0.0401 (2)	−0.00062 (15)	0.00349 (16)	−0.00623 (16)
S2	0.0315 (4)	0.0221 (3)	0.0298 (4)	0.0011 (3)	0.0010 (3)	−0.0023 (3)
S3	0.0348 (5)	0.0492 (6)	0.0952 (8)	−0.0068 (4)	0.0218 (5)	−0.0268 (5)
N4	0.0328 (13)	0.0283 (13)	0.0595 (18)	−0.0020 (11)	−0.0021 (13)	−0.0037 (13)
N6	0.0344 (14)	0.0333 (14)	0.0383 (15)	−0.0027 (11)	0.0053 (12)	−0.0028 (11)
N5	0.0318 (13)	0.0241 (13)	0.0568 (17)	0.0039 (11)	−0.0007 (13)	−0.0033 (12)
C14	0.0492 (18)	0.0220 (14)	0.0329 (16)	0.0044 (13)	0.0047 (15)	−0.0025 (12)
C16	0.0319 (15)	0.0270 (15)	0.0404 (17)	−0.0057 (12)	−0.0011 (14)	−0.0036 (13)
C12	0.0343 (15)	0.0246 (15)	0.0313 (15)	0.0017 (12)	0.0002 (13)	0.0021 (12)
C15	0.0475 (18)	0.0212 (14)	0.0335 (16)	−0.0015 (13)	−0.0060 (16)	−0.0024 (12)
C13	0.0436 (17)	0.0219 (14)	0.0444 (18)	0.0004 (13)	−0.0016 (15)	−0.0017 (13)
C7	0.0388 (17)	0.0244 (16)	0.071 (2)	−0.0027 (13)	−0.0087 (17)	0.0017 (16)
C8	0.045 (2)	0.0370 (19)	0.071 (3)	0.0051 (16)	−0.0147 (19)	−0.0061 (18)
C11	0.0334 (18)	0.041 (2)	0.091 (3)	−0.0019 (16)	0.010 (2)	−0.012 (2)
C10	0.0366 (18)	0.042 (2)	0.103 (4)	0.0095 (16)	−0.015 (2)	0.003 (2)
C9	0.046 (2)	0.047 (2)	0.093 (3)	0.0064 (18)	−0.028 (2)	−0.007 (2)

### Geometric parameters (Å, º)

**Table:**

Cu1—S2	2.2835 (8)	C15—H15A	0.96 (3)
Cu1—S2^i^	2.6491 (8)	C15—H15B	0.96 (4)
Cu1—N6	1.924 (3)	C13—H13A	0.9900
Cu1—C14	2.068 (3)	C13—H13B	0.9900
Cu1—C15	2.095 (3)	C7—H7A	0.9900
S2—C12	1.733 (3)	C7—H7B	0.9900
S3—C16	1.636 (3)	C7—C8	1.490 (5)
N4—C12	1.329 (4)	C8—H8A	0.9900
N4—C7	1.476 (4)	C8—H8B	0.9900
N4—C11	1.476 (4)	C8—C9	1.517 (5)
N6—C16	1.151 (4)	C11—C10	1.494 (6)
Cu1—*Cg*1	1.969	C11—H11A	1.04 (4)
N5—H5	0.856 (10)	C11—H11B	0.91 (5)
N5—C12	1.336 (4)	C10—H10A	0.9900
N5—C13	1.464 (4)	C10—H10B	0.9900
C14—C15	1.351 (5)	C10—C9	1.528 (6)
C14—C13	1.492 (5)	C9—H9A	0.9900
C14—H14	0.98 (4)	C9—H9B	0.9900
			
S2—Cu1—S2^i^	101.98 (3)	H15A—C15—H15B	115 (3)
N6—Cu1—S2	107.15 (8)	N5—C13—C14	114.1 (3)
N6—Cu1—S2^i^	97.44 (8)	N5—C13—H13A	108.7
N6—Cu1—C14	147.71 (13)	N5—C13—H13B	108.7
N6—Cu1—C15	111.75 (13)	C14—C13—H13A	108.7
C14—Cu1—S2	95.68 (9)	C14—C13—H13B	108.7
C14—Cu1—S2^i^	99.83 (9)	H13A—C13—H13B	107.6
C14—Cu1—C15	37.86 (13)	N4—C7—H7A	109.6
C15—Cu1—S2	130.85 (10)	N4—C7—H7B	109.6
C15—Cu1—S2^i^	101.56 (10)	N4—C7—C8	110.3 (3)
Cu1—S2—Cu1^i^	78.02 (3)	H7A—C7—H7B	108.1
C12—S2—Cu1^i^	102.73 (10)	C8—C7—H7A	109.6
*Cg*1—Cu1—S2	113.57	C8—C7—H7B	109.6
*Cg*1—Cu1—S2^i^	101.31	C7—C8—H8A	109.3
*Cg*1—Cu1—N6	129.88	C7—C8—H8B	109.3
C12—S2—Cu1	111.18 (10)	C7—C8—C9	111.8 (4)
C12—N4—C7	123.7 (3)	H8A—C8—H8B	107.9
C12—N4—C11	125.0 (3)	C9—C8—H8A	109.3
C7—N4—C11	111.0 (3)	C9—C8—H8B	109.3
C16—N6—Cu1	177.9 (3)	N4—C11—C10	110.1 (4)
C12—N5—H5	118 (3)	N4—C11—H11A	114 (2)
C12—N5—C13	126.5 (3)	N4—C11—H11B	109 (3)
C13—N5—H5	113 (3)	C10—C11—H11A	101 (2)
Cu1—C14—H14	106 (2)	C10—C11—H11B	113 (3)
C15—C14—Cu1	72.15 (19)	H11A—C11—H11B	109 (3)
C15—C14—C13	123.0 (3)	C11—C10—H10A	109.6
C15—C14—H14	120 (2)	C11—C10—H10B	109.6
C13—C14—Cu1	106.9 (2)	C11—C10—C9	110.4 (3)
C13—C14—H14	115 (2)	H10A—C10—H10B	108.1
N6—C16—S3	179.1 (3)	C9—C10—H10A	109.6
N4—C12—S2	119.9 (2)	C9—C10—H10B	109.6
N4—C12—N5	119.1 (3)	C8—C9—C10	110.5 (3)
N5—C12—S2	121.0 (2)	C8—C9—H9A	109.6
Cu1—C15—H15A	104.7 (19)	C8—C9—H9B	109.6
Cu1—C15—H15B	102 (2)	C10—C9—H9A	109.6
C14—C15—Cu1	69.98 (18)	C10—C9—H9B	109.6
C14—C15—H15A	121.1 (18)	H9A—C9—H9B	108.1
C14—C15—H15B	123 (2)		
			
Cu1—S2—C12—N4	−157.3 (2)	C13—N5—C12—S2	2.0 (5)
Cu1^i^—S2—C12—N4	−75.4 (3)	C13—N5—C12—N4	−177.4 (3)
Cu1^i^—S2—C12—N5	105.2 (3)	C13—C14—C15—Cu1	−98.9 (3)
Cu1—S2—C12—N5	23.3 (3)	C7—N4—C12—S2	−3.8 (5)
Cu1—C14—C13—N5	78.7 (3)	C7—N4—C12—N5	175.6 (3)
N4—C7—C8—C9	−55.6 (4)	C7—N4—C11—C10	−61.3 (4)
N4—C11—C10—C9	57.8 (5)	C7—C8—C9—C10	52.8 (5)
C12—N4—C7—C8	−114.7 (4)	C11—N4—C12—S2	−177.5 (3)
C12—N4—C11—C10	113.1 (4)	C11—N4—C12—N5	1.9 (5)
C12—N5—C13—C14	−62.8 (4)	C11—N4—C7—C8	59.7 (4)
C15—C14—C13—N5	158.0 (3)	C11—C10—C9—C8	−53.6 (5)

Symmetry code: (i) −*x*+1, −*y*+1, −*z*+1.

### Hydrogen-bond geometry (Å, º)

**Table:**

*D*—H···*A*	*D*—H	H···*A*	*D*···*A*	*D*—H···*A*
N5—H5···S3^ii^	0.86 (2)	2.60 (3)	3.375 (3)	151 (3)
C7—H7*B*···S2	0.99	2.48	3.028 (3)	114

Symmetry code: (ii) −*x*+1, *y*+1/2, −*z*+1/2.
